# Open-label placebos reduce test anxiety and improve self-management skills: A randomized-controlled trial

**DOI:** 10.1038/s41598-019-49466-6

**Published:** 2019-09-16

**Authors:** Michael Schaefer, Claudia Denke, Rebecca Harke, Nina Olk, Merve Erkovan, Sören Enge

**Affiliations:** 1 Medical School Berlin, Calandrellistr. 1-9, 12447 Berlin, Germany; 2 Department of Anesthesiology and Intensive Care Medicine, Charité – Universitätsmedizin Berlin, Berlin, Germany

**Keywords:** Psychology, Randomized controlled trials

## Abstract

Test anxiety is a condition in which people experience extreme distress and anxiety before and in test situations. It affects up to 40 percent of all students. Conventional treatment includes both medication and psychotherapy, but studies also demonstrated that placebos affect anxiety symptoms. Although in the traditional understanding placebos need to be administered in a concealed way, intriguing new studies report that open-label placebos can be effective. Since prescription of fake pills involves ethical problems, open-label placebos may provide important new treatment possibilities. Here we report results of a pilot study examining whether open-label placebos may reduce test anxiety and improve self-management skills. 58 students participated in a two-group randomized controlled trial. Two weeks before an exam at the university participants received open-label placebos or no pills (control group). Participant – provider relationship and amount of contact time was held similar for all groups. After two weeks we found that test anxiety and self-management abilities (skills and resources) of the open-label placebo group were more improved than in the control group. Thus, our results seems to indicate that open-label placebos may reduce test anxiety and enhance self-management skills in students.

## Introduction

Test anxiety is a condition in which people experience extreme distress and anxiety before and in test situations. During adolescence, many students report such anxiety problems. According to Cassady, test anxiety affects up to 40 percent of all students^1,2^. For example, in Germany 13% of all first-year undergraduate students seek counseling services for test anxiety^3^. Anxiety in test situations has different facets, including affective (e.g., a nervous feeling of excitement), physiological (e.g., heart rate, sweating), motivational (e.g., avoidance tendencies) and cognitive components (e.g., concerns for failure)^4^. It has been shown that test anxiety weakens the ability to perform cognitively challenging tasks and reduces motivation, although test anxiety can also be beneficial for motivation, for example, when students try to avoid errors more rigorously^1^.

Conventional treatment of test anxiety includes both medication and psychotherapy^1^. In addition, an increasing number of students seem to use neuro enhancement (taking prescription stimulants) to reduce test anxiety^5^. However, studies also demonstrated that placebos may reduce anxiety^6^. Numerous studies demonstrated placebo effects for many different conditions^7^. Unfortunately, the administration of placebos is considered unethical with therapeutic purposes because concealment is thought to be necessary and would therefore cause problems with respect to informed consent and trust.

However, several randomized controlled studies examined effects of placebos without concealment (open-label placebo, OLP) and questioned whether deception of placebo is necessary to elicit placebo effects. For example, Kaptchuk *et al*. administered OLPs to patients with irritable bowel syndrome^8^. Patients who took the OLPs had significantly improved irritable bowel syndromes, higher mean global improvement scores, and better quality of life scores compared with a no-treatment control group. Hence, although the patients knew that they received placebos, they demonstrated a placebo effect. Similar results have been reported, for example, for depression, chronic low-back pain, cancer related fatigue, migraine-induced headaches, and allergic rhinitis^9–16^. Given that OLPs do not include ethical problems such as undermining trust in the patient-physician relationship or violating the principle of informed consent, OLPs may provide important new treatment possibilities^17^.

The current study aimed to examine whether OLPs improve test anxiety and self-management skills more than a no-treatment control group. We conducted a randomized controlled trial including student participants two weeks before they underwent an exam at the university. In the OLP group participants received placebos without deception, while the control group received no pills. Two weeks later we tested whether test anxiety, self-management skills and resources, and quality of life had improved as an effect of OLPs.

## Materials and Methods

### Participants

**Figure 1:**
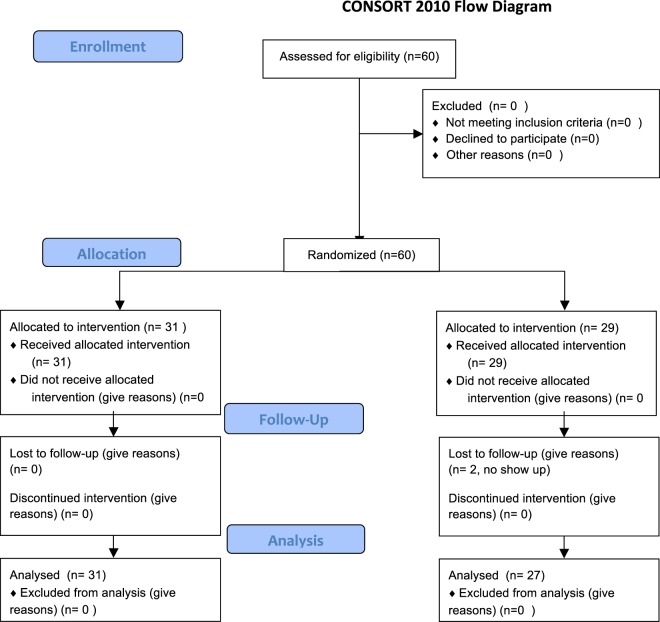
Flow diagram of patient’s enrollment.

58 students (mean age 22.9 ± 2.0 years, 50 females) took part in the study and provided written informed consent (see Flow chart, Fig. 1). The study was approved by the ethical board of the German Psychological Society (Deutsche Gesellschaft für Psychologie, DGPs) and registered at the German clinical trials register (DRKS00013621, registered on 30/01/2018). The authors assert that all procedures contributing to this work comply with the ethical standards of the relevant national and institutional committees on human experimentation and with the Helsinki Declaration of 1975, as revised in 2008.

Participants were recruited via flyers at the local university and social media. Inclusion criteria were age (18–60 years), being a student at a university, and having an exam at the end of the semester. Exclusion criteria included pregnancy, diabetes and any psychiatric or neurological diseases. Participants’ characteristics are shown in Table 1.

**Table 1:** Demographic and baseline characteristics of participants.

Characteristic	Open label placebo	Control
N	31	27
Age (mean ± SD in years)	22.3 ± 2.3	23.5 ± 3.4
Females/Males	25/6	25/2
Grade Point Average (mean ± SD)	2.1 ± 0.5	2.1 ± 0.5
Test anxiety (PAF)	45.71 ± 0.36	49.00 ± 0.95
Self-management abilities (FERUS)	157.48 ± 28.82	164.63 ± 21.26
Resource Change Motivation (FERUS)	32.7 ± 9.7	36.1 ± 9.2
Resource Perceived Social Support (FERUS)	43.1 ± 7.94	43.8 ± 5.66
Quality of Life (SF-36): mental sum score	40.57 ± 11.56	41.12 ± 9.59
Quality of Life (SF-36): physical sum score	53.66 ± 7.59	50.92 ± 7.57

### Study design

58 students were allocated to a two week randomized controlled trial comparing open-label placebo to no-treatment controls. For both groups participant-provider interaction and amount of contact time was held similar. After two weeks (before taking the exam) we tested whether test anxiety and self-management abilities had changed.

Sample size was based on previous research^18^. Considering an alpha error probability of 0.05 and an estimated effect size of d = 0.8, we calculated 26 participants per cell as necessary for a desired power of 0.80.

### Procedure

During the first visit all participants were briefed in the same way (as described in^14^). They were explained that placebos are inactive substances and that they contain no medications, but placebo effects may still be powerful. They were told that the body may automatically respond to taking placebo pills, like Pavlov’s dogs that salivated when they heard the bell. A positive attitude may be helpful for the placebo effect, but is not necessary. Last, they were told that those participants who were in the placebo group needed to take the placebos faithfully^8^. These four statements are identical to the instruction used in previous studies on open-label placebos^8,9,13^.

Subsequently to this information participants completed questionnaires in order to assess test anxiety (German Test Anxiety Inventory, PAF^19^), quality of life (SF-36^20^), and self-management abilities (FERUS^21^). The PAF is a multi-faceted measure of test anxiety and has been used to detect anxiety levels in secondary schools and college students. It consists of 20 items and 4 subscales (worry, emotionality, interference, lack of confidence). The SF-36 assesses quality of life in patients and healthy individuals. It addresses different health concepts and asks, for example, for limitations in physical or social activites because of health or emotional problems, bodily pain, psychological distress and well-being, and general health perceptions. Self-management abilities were measured using the FERUS, which provides a global score for self-management skills and two additional measures (resource change motivation and perceived social support). The global score for self-management skills consists out of five scales (coping, self-efficacy, introspection, hope, and self verbalization).In addition, we asked all participants for the average grade of their previous exams.

Then an opaque envelope was opened, revealing the participant’s randomized assignment. Participants randomized to the OLP group were given a white tube, labeled with the logo of the local university and marked with: “Placebo pills (28), take one in the morning and one before night, for 14 days”. Placebo pills contained sugar, wheat- and cornstarch, and glucose syrup (identical to^14^). The pills were white, round, and the size was about 4 mm. Participants were instructed to swallow the pills, not to chew or suck them. Participants in the control group received no pills at all, but were reminded of the importance of the control group. After two weeks, on the days before taking the final exam, we invited the participants for a second visit, in which we again assessed test anxiety, self-management abilities, and quality of life. The research assistant was blind to the group assignments of the participants. Finally, participants of the control group were asked about possible feelings of disappointment to be assigned to the control group.

Last, we contacted all subjects after their exam and asked them about their examination grade.

### Statistical analysis

The main outcomes were changes from pre to post in test anxiety (PAF) and self-management-skills (FERUS). A further outcome was quality of life (SF-36). Outcomes were measured at baseline and after two weeks (before the exam). Baseline data were examined for differences between groups using independent t-tests.

We computed separate analyses of variance (ANOVA, two factors: time and group) to test differences for these outcomes. Significant changes in test anxiety, self-management abilities, and quality of life were then further analyzed using paired sample t-tests. Cohen’s d was used to assess the power of effects.

The health survey for measuring quality of life (SF-36) results in a physical and a mental sum score. The first one refers to quality of life with respect to the body state, whereas the last one reports mental or emotional quality of life. We calculated separate ANOVAs based on these measures.

Furthermore, we calculated correlations (Pearson) in order to exam whether a possible improvement in test anxiety is accompanied with changes in self-management skills or quality of life.

Last, we computed Pearson’s correlations in order to investigate possible relationships between improvements in test anxiety or self-management skills, respectively, with the performance in the final exam.

We used the SPSS software package for all statistical analysis (IBM Corp., Armonk, NY, USA). P values of < 0.05 were considered as significant.

## Results

Baseline scores of the groups (PAF, FERUS, SF-36, grade point average) before starting the trial were not different (all p’s > 0.1).

**Figure 2:**
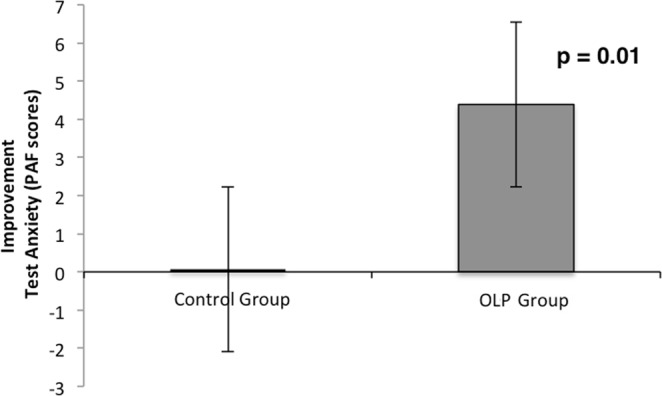
Improvement of test anxiety (PAF) for OLP and control group (mean and standard error) after two weeks. Results indicate a significant improvement for the OLP group from pre to post. For the control group no significant difference from pre to post was found.

Results revealed significant interactions between group and time for test anxiety (ANOVA with factors group and time, F (1,56) = 4.23, p < 0.05, η^2^ = 0.07). Post hoc t-tests demonstrated that test anxiety in the OLP group was significantly reduced (change score: 4.39; t(30) = 2.61, p ≤ 0.01, two-sided, Cohen’s d_z_ = 0.5). In contrast, the control group showed only a very small and non-significant reduction (change score: 0.07, p > 0.10, see Fig. 2 and Table 2).

**Table 2:** Changes on outcome measures at 2-week endpoint (mean ± SD).

	Open-label placebo	Control
Test Anxiety (PAF scores)	4.39 ± 9.35	0.07 ± 6.0
Self-Management Abilities (FERUS)	15.77 ± 31.71	2.48 ± 15.46
Resource Change Motivation (FERUS)	1.19 ± 5.37	−1.81 ± 5.88
Resource Perceived Social Support (FERUS)	1.68 ± 7.60	−0.96 ± 6.94
Quality of Life (SF-36): mental sum score	3.50 ± 12.69	−0.77 ± 8.89
Quality of Life (SF-36): physical sum score	−0.6 ± 9.39	0.5 ± 8.67
Examination grade	2.1 ± 0.9	1.9 ± 0.6

**Figure 3:**
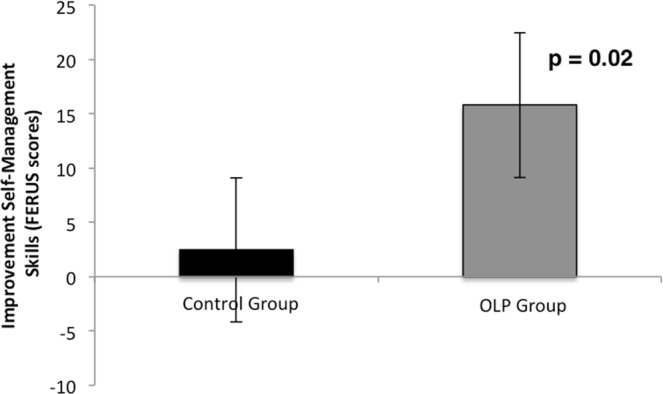
Changes of self-management abilities (FERUS) for OLP and control group (mean and standard error). Results demonstrate a significant improvement from pre to post for the OLP group, but not for the control group.

**Figure 4:**
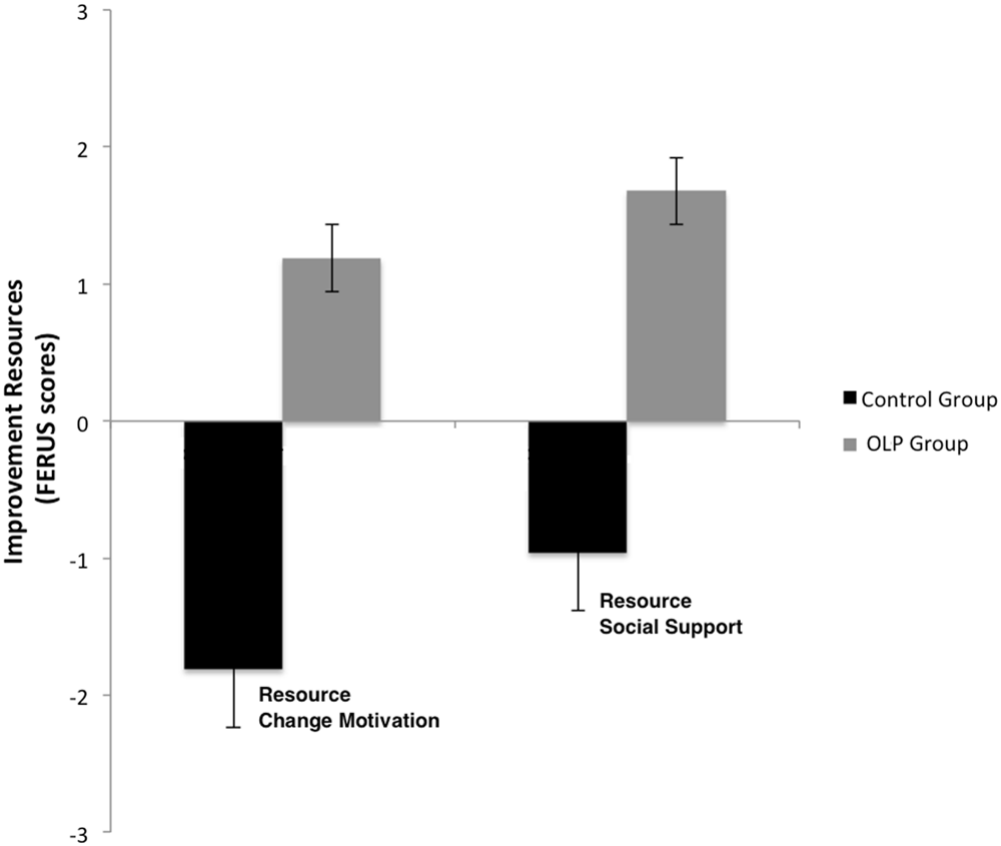
Changes of resources (FERUS: change motivation and perceived social support) for OLP and control group (mean and standard error). Results demonstrate an improvement in change motivation from pre to post for the OLP group, whereas for the control group change motivation was reduced. Results for perceived social support showed a similar pattern, but failed to reach the level of significance.

Further analysis revealed an interaction between group and time for changes in self-management skills and resources (ANOVA with factors group and time; skills: F(1,56) = 3.93, p ≤ 0.05, η^2^ = 0.07; resource change motivation: F(1,56) = 4.15, p ≤ 0.05, η^2^ = 0.07; resource social support: p = n.s., see Figs 3 and 4). Post hoc t-tests showed that self-management skills in the OLP group were significantly enhanced (change score: 15.78; t(30) = −2.70, p ≤ 0.01, Cohen’s d_z_ = 0.63), whereas the control group showed only a small and non-significant change (change score: 2.48, p > 0.10) (see Fig. 3).

The improved self-management skills were linked to the reduction in test anxiety (Pearson correlation, OLP group: r = 0.55, p < 0.001, control group: r = 0.39, p < 0.01). In addition, the improvement of self-management abilities (resource change motivation) in the OLP group was positively correlated with exam results, suggesting that the improvement in change motivation here resulted in better exam results (OLP: r = 0.34, p = 0.06; control: r = 0.16, p = 0.40).

The outcome measure quality of life (mental sum score of the SF-36) was enhanced in the OLP group, but failed to reach the level of significance (OLP: change score 3.50; control: change score −0.77). The improvement in quality of life in the OLP group was significantly correlated with the reduction of test anxiety (r = 0.52, p = 0.003; control: p > 0.10) and with the change in self-management skills (r = 0.61, p < 0.001; control: p > 0.10). The physical sum score revealed no significant effects.

None of the participants of the control group stated to be disappointed to be assigned to the control group. There were no adverse effects in the placebo group.

## Discussion

This study showed that open-label placebos reduced test anxiety and improved self-management skills (and perceived resources) before an academic exam better than a control group with the same quality of interaction with advisers. Moreover, the improvement of the self-management resources in the OLP group was positively linked to the exam results. Thus, the results suggest that an OLP treatment may improve self-management abilities and test anxiety.

Our results are in line with previous OLP studies. While previous studies demonstrated effects in various diseases (e.g., in patients with depression or chronic back pain^8,10,13,16^), our results suggest that even healthy participants may profit from OLPs by preventing or reducing test anxiety and improving self-management skills.

How did placebos improve test anxiety and self-management abilities in our study? The way OLPs might work remains to be cleared^18,22,23^. OLP effects are explained by classical conditioning, suggesting that placebos may retrieve a pharmacological memory^24^. Another explanation refers to embodied cognition. The theory of embodiment states that mind and world interact via the body and thereby may influence our cognitions^25^. It is important to note that this explanation does not need any specific conditioning procedure. Furthermore, the patient - healthcare provider relationship might explain OLP effects. Interaction of the patient with the healthcare provider often results in feeling socially supported, which then in turn might affect the individual health status. All these processes could also work together to cause beneficial effects.

Several limitations of this study should be noted. Most importantly, the sample size was relatively small. Therefore, it should be classified as a pilot study. Thus, we have to be careful when drawing conclusions out of these data. Future studies are needed to replicate the results with a bigger sample size. Moreover, trial duration was short and we do not know whether there are long-term effects. Furthermore, future studies should include a covered placebo condition. In addition, a potential confounder in our study design is the time when we informed the participants about the potential power of placebos. It would have been better to provide this information after baseline assessment, not prior. Another potential confounder may be that only one group received the treatment. The outcomes for the control group may have been different if they had known they would also get the placebos (e.g., in a follow-up phase for another exam).

This study is the first to suggest that OLPs may improve self-management skills and test anxiety. Further research needs to be done, but we think that the present results are promising.

### Clinical Trial Registration Number

German Clinical Trials Register, DRKS00013621.
